# Perichondritis in a Six-Week-Old Pediatric Patient

**DOI:** 10.7759/cureus.88528

**Published:** 2025-07-22

**Authors:** Jacob J Dunn, Thiagarajan Nandhagopal, Audrey Nguyen, Michael Q Tran

**Affiliations:** 1 Pediatrics, Kern Medical Center, Bakersfield, USA

**Keywords:** auricular infection, infant, pediatric case, perichondritis, pseudomonas aeruginosa

## Abstract

Perichondritis is an uncommon infection of the auricular cartilage and surrounding tissue, typically seen in older children and adults following trauma or piercing. It is rarely reported in infants, making early recognition and management in this age group essential to prevent complications such as cartilage necrosis and auricular deformity. A six-week-old previously healthy infant presented with erythema, swelling, and tenderness of the left auricle without a history of trauma, insect bite, or recent upper respiratory infection. Initial evaluation revealed signs consistent with perichondritis. Empiric antibiotic therapy was initiated with intravenous anti-pseudomonal and anti-staphylococcal coverage. The patient responded well to intravenous antibiotics, followed by oral therapy, with complete resolution of symptoms and no long-term deformity or recurrence noted at two-week follow-up. Perichondritis in infants is rare and may present without a clear precipitating factor. Prompt diagnosis and appropriate antibiotic therapy are crucial in preventing complications. This case highlights the importance of including perichondritis in the differential diagnosis of auricular swelling in infants, even in the absence of trauma or piercing.

## Introduction

Perichondritis, an infection of the cartilaginous tissue and surrounding perichondrium, is a rare condition in the pediatric population, particularly in neonates and infants [1,2]. While it most commonly affects the pinna of the ear in older children and adults, often secondary to trauma, surgery, or piercing, it is exceptionally uncommon in the first weeks of life due to the limited exposure to such risk factors [1,3]. In infants, perichondritis may arise from unique etiologies, including congenital anomalies, iatrogenic injury, or bacterial seeding from systemic infection, posing diagnostic and therapeutic challenges [2]. Notably, abnormal laboratory findings are often absent in infantile perichondritis, further complicating diagnosis. This case report presents a rare instance of perichondritis in a six-week-old infant, highlighting the clinical presentation, diagnostic approach, and management strategy employed. By documenting this unusual occurrence, we aim to contribute to the sparse literature on perichondritis in this age group and underscore the importance of early recognition and intervention to prevent long-term complications, such as cartilage destruction or deformity.

## Case presentation

A six-week-old infant born at 38 weeks gestation with a history of congenital syphilis treated with intravenous penicillin postnatally presented to the emergency department (ED) with a three-day history of worsening right ear lobe swelling and redness. The patient’s mother reported that the symptoms began after the infant possibly scratched herself, with progressive erythema, swelling, and fussiness upon touching the affected area. The infant was otherwise well, tolerating three to four ounces of formula every four hours without emesis, producing six wet diapers and one to two bowel movements daily, and exhibiting no fever, respiratory symptoms, or additional rashes. There was no history of trauma, otorrhea, or sick contacts.

Upon examination in the ED, the patient was afebrile with normal vital signs. Physical examination revealed moderate swelling and erythema of the right pinna with tenderness on palpation, but no discharge from the ear canal. Tympanic membranes were normal bilaterally, and the remainder of the exam was unremarkable, with no signs of systemic illness. Laboratory evaluation on admission, summarized in Table 1, demonstrated a normal white blood cell count, C-reactive protein, and procalcitonin. No diagnostic imaging was obtained. Given the patient's presentation, along with initial laboratory findings, the differential diagnosis included perichondritis, cellulitis, otitis externa, contact dermatitis, and syphilitic skin manifestations due to the history of treated congenital syphilis. However, the specific involvement of the auricular cartilage, absence of ear canal discharge, and normal inflammatory markers (Table 1) strongly favored perichondritis. 

**Table 1:** Initial laboratory findings on admission

Parameters	Patient Values	Reference Range
White Blood Cell Count	10.1 × 10³/μL	5.0–19.5 × 10³/μL
C-reactive Protein	<0.3 mg/L	<3.0 mg/L
Procalcitonin	0.08 ng/mL	<0.5 ng/mL

The patient was initiated on IV ceftazidime 50 mg/kg q12h on hospital day zero to cover gram-negative bacteria, specifically Pseudomonas, along with topical mupirocin applied three times daily to the external right ear. On hospital day one, examination showed persistent erythema with new yellow scaling in the right ear canal, prompting the addition of IV clindamycin 10 mg q6h. On hospital day two, yellow-crusted lesions were also noted on the hairline and eyelids. By hospital day three, the right ear erythema had improved with no tenderness, though scaling persisted. Blood cultures were negative at 48 hours, and a wound culture revealed rare mixed skin flora. Ceftazidime was continued for a seven-day course, while IV clindamycin was discontinued on hospital day four after three days and switched to oral clindamycin 75 mg qd for two days. Topical mupirocin was maintained through hospital day six. The patient continued to feed well, with normal stooling and voiding throughout admission. By hospital day five, the right ear erythema was fading, and on hospital day six, the patient was discharged after completing seven days of IV ceftazidime, four days of IV clindamycin, and two days of oral clindamycin. 

At a well-child visit 15 days post-discharge, the now two-month-old infant was breastfeeding well, weighed 12 pounds 10 ounces, and was meeting developmental milestones. The rash had completely resolved and the mother reported no further concerns.

## Discussion

Perichondritis is an uncommon infection of the auricular cartilage and surrounding perichondrium, most frequently associated with trauma, piercings, or surgical interventions in older children and adults [1,3]. In neonates and young infants, the condition is exceedingly rare, with few documented cases in the literature [2]. This report presents a notable instance of perichondritis in a six-week-old infant with a history of congenital syphilis, who developed auricular inflammation. The clinical course underlines several key considerations in the diagnosis and management of this rare medical condition in very young patients.

The early signs of perichondritis are erythema, swelling, and tenderness of the pinna and are nonspecific and may be mistaken for cellulitis or otitis externa [2]. In neonates and young infants, such presentations warrant careful evaluation, given their limited ability to localize symptoms and the risk of rapid progression [1]. Notably, the patient in this case remained afebrile and systemically well-appearing, with only localized findings and normal inflammatory markers (Table 1), making the diagnosis less obvious. The absence of fever or leukocytosis should not preclude a high index of suspicion when localized cartilage inflammation is observed [2,3]. This underscores a key diagnostic challenge in infants: objective laboratory findings may be unremarkable despite a clinically significant infection. Compared to more common cases of perichondritis in adolescents or adults, which often follow trauma or piercing and may present with fluctuance, purulent drainage, or systemic signs [1,3], this infant's presentation was subtle and without clear inciting factors. The localized erythema and tenderness without systemic illness or otorrhea helped differentiate perichondritis from otitis externa or cellulitis [2]. This is in contrast to typical cases, which supports the need for clinician vigilance when encountering auricular swelling in young infants. Photographic imaging could not be included. This limitation is noted here and should be considered in future reports where feasible, as visual documentation can support pattern recognition in rare pediatric conditions. The patient's history of congenital syphilis, treated with intravenous penicillin postnatally, was considered in the differential diagnosis. However, neither the treated syphilis nor its penicillin therapy is likely to have predisposed the patient to perichondritis, as there is no reported association between penicillin treatment and increased susceptibility to localized bacterial infections like perichondritis [1,2]. The clinical presentation, including a reported minor scratch as a potential inciting event, and the rapid response to anti-pseudomonal and anti-staphylococcal antibiotics suggest a primary bacterial etiology, consistent with typical pathogens such as Pseudomonas aeruginosa and Staphylococcus aureus [1,2]. The absence of fever, leukocytosis, or syphilitic sequelae further supports that the prior syphilis and its treatment were unrelated to the current symptoms. 

Pseudomonas aeruginosa is the most commonly implicated pathogen in perichondritis, particularly in association with piercings or trauma; however, Staphylococcus aureus and other skin flora are also frequently isolated [2-4]. In this case, although cultures were non-specific, revealing only rare mixed skin flora, the empiric use of intravenous ceftazidime provided appropriate coverage for Pseudomonas, while clindamycin was added for gram-positive and anaerobic organisms [4]. The decision to broaden antibiotic coverage after new scaling and crusting emerged was prudent and likely contributed to the patient’s favorable clinical outcome. Topical mupirocin was also appropriately utilized for localized antimicrobial effects [3].

Although there are no standardized treatment guidelines for neonatal perichondritis, the seven-day course of IV ceftazidime, along with a short course of IV followed by oral clindamycin, aligns with management strategies for other soft tissue infections in this age group [3,4]. Close inpatient monitoring ensured early detection of treatment response and any systemic progression. The resolution of symptoms without cartilage deformation or relapse on follow-up highlights the importance of early intervention [1,2]. This case reinforces that prompt initiation of appropriate antibiotics can prevent long-term complications, such as cartilage necrosis or auricular deformity. 

## Conclusions

This case report describes a rare presentation of perichondritis in a six-week-old infant, an age group where such infections are exceptionally uncommon due to the absence of typical risk factors like trauma or piercing. The successful resolution of symptoms with prompt empiric antibiotic therapy, including intravenous ceftazidime and clindamycin followed by oral clindamycin, underscores the importance of early recognition and tailored antimicrobial management in preventing complications such as cartilage necrosis or auricular deformity. The absence of systemic symptoms and normal inflammatory markers in this case highlights the need for a high index of suspicion for perichondritis when evaluating localized auricular inflammation in infants, even without clear precipitating factors. This report adds to the limited literature on neonatal perichondritis and emphasizes the critical role of multidisciplinary care and close follow-up in achieving favorable outcomes. Pediatric clinicians should consider perichondritis in the differential diagnosis of auricular swelling in young infants, even in the absence of objective diagnostic markers, to ensure timely intervention and optimal recovery.
